# Basic Life Support Awareness, Knowledge, and Attitudes Among a Self-Selected Online Sample in the Aseer Region, Saudi Arabia: An Exploratory Cross-Sectional Survey

**DOI:** 10.3390/healthcare14193311

**Published:** 2026-10-04

**Authors:** Ibrahim Tawhari, Shahad Alshehri, Ahad Shafi Alaklabi, Raghad Jubran Alqahtani, Afaf Haif Qahtani, Baylasan Meraie E. Alqahtani, Reema Abdullah S. Alsharif, Danah Mansour Almutib, Ghala Ali Alshehri

**Affiliations:** 1 Department of Internal Medicine, College of Medicine, King Khalid University, Abha 62521, Saudi Arabia; 2 College of Medicine, King Khalid University, Abha 62521, Saudi Arabia; shahadshehri39@gmail.com (S.A.); ahadshafi20@outlook.com (A.S.A.); raghad-2442@hotmail.com (R.J.A.); dr.afafhq@yahoo.com (A.H.Q.); bylasan.alg@gmail.com (B.M.E.A.); alsharifreemay@gmail.com (R.A.S.A.); danah.almutib@gmail.com (D.M.A.); 3 Nursing College, King Khalid University, Abha 62521, Saudi Arabia; alshehrrighala@gmail.com

**Keywords:** basic life support, cardiopulmonary resuscitation, CPR training, public awareness, Saudi Arabia, cross-sectional study, Aseer Region, emergency response

## Abstract

**Background/Objectives:** Basic life support (BLS) and cardiopulmonary resuscitation (CPR) are essential emergency interventions. This exploratory study described BLS awareness, reported training, self-assessed and objectively scored CPR knowledge, and attitudes in a self-selected online sample of adults in the Aseer Region, Saudi Arabia, and examined how these constructs related to one another; it was not designed to estimate regional prevalence. **Methods:** A cross-sectional online survey (April–June 2025) used convenience recruitment through social media. The study-specific questionnaire was expert-reviewed and pilot-tested but not psychometrically validated; the five technical items (Kuder–Richardson 20 = 0.59) were analyzed individually. Analyses were primarily descriptive; comparisons, including a multivariable logistic regression, were exploratory. **Results:** Of 364 respondents, 87.1% were female, 49.5% were students, and 67.9% held a bachelor’s degree. Overall, 90.9% had heard of BLS, 47.3% reported CPR training, and 45.1% reported knowing how to perform CPR; 27.5% to 57.1% selected the keyed answer on individual objectively scored items. Only 20.7% of those reporting knowing how to perform CPR selected all four keyed answers. Respondents reporting CPR training selected keyed answers more often on every objectively scored item, most markedly for the 30:2 compression-to-ventilation ratio (50.6% vs. 18.2%), yet only 41.3% of them selected the “at least 100 compressions/min” option. Measured sociodemographic characteristics explained little of the variation in reported training (McFadden pseudo-R^2^ = 0.11). **Conclusions:** In this demographically imbalanced sample, self-reported awareness and self-assessed ability to perform CPR were often not matched by objectively scored knowledge. Associations with reported training may reflect who seeks training rather than training effects, and item wording may have influenced responses. The shared dataset and identified instrument weaknesses can inform representative studies using validated measures and objective skills assessment.

## 1. Introduction

Basic life support (BLS) is a critical component of emergency medical care that encompasses a set of life-saving techniques, including cardiopulmonary resuscitation (CPR), airway management, and the use of automated external defibrillators (AEDs). These skills are designed to sustain life during cardiac arrest and other medical emergencies until advanced professional care becomes available [1].

Cardiac arrest remains a major global health concern and is one of the leading causes of preventable death worldwide. Survival rates following out-of-hospital cardiac arrest (OHCA) are critically influenced by the speed and quality of bystander CPR initiation. In a Swedish national cohort of 30,381 witnessed arrests, 30-day survival was 10.5% when CPR was started before the arrival of emergency medical services, compared with 4.0% when it was not (*p* < 0.001) [1]. However, the effectiveness of bystander CPR is closely tied to community-level awareness, knowledge, and willingness to act in an emergency [2].

Despite the well-established importance of BLS, multiple studies have demonstrated that awareness and practical knowledge of CPR remain limited in various communities, particularly in low- and middle-income countries and in regions with limited formal emergency medical services (EMS) infrastructure [3,4]. In Saudi Arabia, several studies have examined BLS awareness across different regions, revealing notable disparities in knowledge and training uptake [5,6].

These findings are consistent with international evidence, in which inadequate BLS knowledge and skills among the general public are frequently reported, and barriers such as fear of causing harm, lack of training, and low confidence in performing CPR continue to hinder effective bystander response [7,8]. Previous studies have also shown that sociodemographic factors, including age, gender, educational level, and occupational background, are associated with both knowledge and attitudes toward BLS [9,10].

Prior Saudi surveys indicate geographic variation in reported BLS awareness and training, but valid regional estimates require representative sampling and comparable validated measures. A search of PubMed, Scopus, and Google Scholar through June 2025, combining “basic life support”, “cardiopulmonary resuscitation”, “awareness”, “knowledge”, and “attitude” with Saudi regional identifiers, identified studies from Riyadh, Jeddah, Jazan, Al-Qassim, Al-Majma’ah, and Ha’il [2,7,9,11,12,13], but none specifically sampling Aseer residents. Because the present study relied on convenience sampling and a study-specific questionnaire, it was designed as an exploratory first step rather than as a source of regional estimates.

Studies in this field often report several distinct constructs under the general heading of BLS awareness or knowledge. In this study, awareness denotes self-reported familiarity with BLS (having heard of it); training denotes self-reported receipt of instruction; self-assessed knowledge denotes respondents’ own judgment of what they know, such as reporting that they know how to perform CPR; objectively scored knowledge denotes selection of the guideline-concordant option on single-best-answer technical items; and attitudes denote reported willingness to perform CPR and concerns about doing so. These constructs are related but not interchangeable: people may have heard of BLS without having been trained, may rate their own knowledge more favorably than their answers to specific items support, and may answer knowledge items correctly without being able to perform CPR. Previous Saudi surveys have described limited practical knowledge despite awareness [11,14] and have often summarized knowledge as composite scores [2,11,14], which can conceal differences among items and depend on the internal consistency of the items combined. Examining these constructs separately within the same respondents, at the level of individual items, can show where the gaps between awareness, training, self-assessment, and knowledge lie, and which specific knowledge components differ with training.

Therefore, this exploratory study aimed to describe BLS awareness, reported training, self-assessed and objectively scored CPR knowledge, and attitudes among adults who participated in an online convenience sample in the Aseer Region; to compare self-assessed with objectively scored knowledge; and to examine, as hypothesis-generating analyses, the associations of reported CPR training with measured sociodemographic characteristics and with item-level knowledge. Its findings are intended to identify hypotheses, educational gaps, and instrument weaknesses for confirmation in representative studies using validated measurement tools.

## 2. Materials and Methods

A cross-sectional, questionnaire-based online survey was conducted in the Aseer Region, Saudi Arabia, between April and June 2025, following ethical approval, and is reported with reference to the Checklist for Reporting Results of Internet E-Surveys (CHERRIES) [15]. Eligible participants were adult residents (aged ≥18 years) of the Aseer Region. No sampling frame of regional residents was available, so participants were recruited by convenience sampling: an anonymous, self-administered electronic questionnaire was distributed through public social media platforms and community networks and could be completed by any eligible resident who chose to open the link. Because the invitation was shared openly rather than sent to a defined list, the number of people who saw it could not be determined and a conventional response rate cannot be calculated. A minimum recruitment target of 315 was set pragmatically using the single-proportion formula *n* = Z^2^*p*(1 − *p*)/d^2^, with Z = 1.96, an expected CPR-training prevalence of 28.7% from a comparable Saudi survey [6], and d = 0.05. Because this formula assumes probability sampling, it served only to set a recruitment target; it does not confer the stated precision on estimates from a convenience sample, and no population-level margin of error is claimed.

The questionnaire was hosted on Google Forms (Google LLC, Mountain View, CA, USA). The consent form was presented on the first page, and respondents could proceed only after recording their agreement. Items were presented in the five sections described below; single-response items were set as required fields, and conditional follow-up items (for example, type of training and training location) were displayed only to respondents to whom they applied. The platform records only submitted questionnaires; therefore, the number of people who viewed or opened the questionnaire without submitting it was unavailable, and the view, participation, and completion rates recommended by CHERRIES could not be calculated. Because the survey was anonymous and collected no identifiers, no cookies, Internet Protocol (IP) address checks, or log-in requirements were used to prevent repeat participation. Submitted questionnaires were screened for affirmed consent, eligibility, and completeness before analysis. As an additional quality-control step, all 366 submitted records were screened for identical response patterns across every questionnaire item; two pairs of records with identical answers to all items, including age and gender, were identified and considered duplicate submissions, and the later submission of each pair was removed, leaving 364 respondents for analysis. The coding of the multiple-response items was also checked against the recorded text responses; five respondents whose only answer to the willingness item was the drug-use option had not been coded because of a variant wording of that option, and they were recoded accordingly. The Supplementary Dataset (Table S1) reflects both corrections, and item-level non-response is reported in Section 2.1.

A study-specific questionnaire was assembled from items used in comparable Saudi and regional BLS awareness surveys [5,6,11], with technical CPR items based on adult BLS recommendations (a 30:2 compression-to-ventilation ratio, 100–120 compressions/min, and a depth of 5–6 cm) that are retained in the current guidelines [16]; it was not a previously validated instrument. The questionnaire comprised five sections: (1) sociodemographic characteristics; (2) BLS/CPR awareness and prior exposure; (3) five technical CPR items (compression–ventilation ratio, compression site, rate, depth, and the AED item); (4) attitudes and practices concerning sudden death and CPR; and (5) perceived barriers to performing CPR. Specialists in internal medicine and emergency care reviewed the items for clarity, relevance, and cultural appropriateness, after which wording was revised. A small pilot group of adult residents then assessed comprehensibility and completion time; pilot responses were excluded from the analysis. Pretesting did not include cognitive interviewing or review by community stakeholders, and these procedures do not constitute psychometric validation (Section 4.7).

Table 1 maps each construct examined to its operational definition and questionnaire items. Training was measured by two self-reported items: a general item on BLS training in the awareness section, followed by questions on its type and frequency, and an item in the CPR section asking about training on “this topic”, which directly followed the question on knowing how to perform CPR and is referred to here as CPR training; the latter was used as the training variable in all comparisons. Self-assessed knowledge comprised the yes/no item on knowing how to perform CPR and the AED item, which asked what respondents knew about the device and offered only “Yes” and “No, I don’t know” as responses; the AED item therefore records self-reported familiarity rather than tested knowledge. Objectively scored knowledge comprised four single-best-answer items, each keyed to the option closest to current adult recommendations [16]: 30:2 for the ratio, the middle of the chest for the compression site, “at least 100 times/minute” for the rate (no option matched the recommended 100–120/min), and 5–6 cm for the depth. The five technical items (the four objectively scored items and the AED item) had a Kuder–Richardson 20 (KR-20) coefficient of 0.59, and the four objectively scored items alone had a KR-20 of 0.54, both below the commonly used 0.70 threshold, indicating that the items do not form an internally consistent scale. The items were therefore analyzed individually, and no composite knowledge score was used for inference; the number of respondents selecting all four keyed answers is reported descriptively only (Section 2.1). The warning-sign item combined myocardial infarction symptoms with cardiac arrest features; it was reported descriptively only and was not included among the knowledge items or in any inferential analysis.

Participants reported prior experience with sudden death, willingness to perform CPR for recipients of differing social proximity, and concerns about performing CPR. Five willingness categories used neutral relational descriptors (a family member, a friend, a neighbor, a young person at the gym, and a stranger at a shopping center). Two further categories, adapted from prior studies of bystander willingness [4,17,18], described the recipient with stigmatizing labels (“dirty and unclean” and drug use); these items formed part of the complete questionnaire approved by the Research Ethics Committee (Section 2.2). The authors do not endorse these labels and consider them inappropriate; responses to these two items are reported in Section 3.2 for transparency only and are not interpreted (Section 4.3). Training location was collected from participants reporting CPR training, and number of sessions from those reporting BLS training.

### 2.1. Statistical Analysis

Descriptive statistics were used to summarize all variables. Categorical variables were expressed as frequencies and percentages. Continuous variables (age) were reported as the mean ± standard deviation (SD) and as the median with interquartile range (IQR). The normality of the continuous data was assessed using the Shapiro–Wilk test, which indicated a non-normal distribution; nonparametric tests were therefore used for group comparisons. In the cross-tabulations of sociodemographic characteristics against receipt of CPR training (Section 3.4), percentages are reported within each sociodemographic category (row percentages); in the cross-tabulations of knowledge and practice items against training status (Section 3.5), percentages are reported within each training group. Self-assessed and objectively scored knowledge were compared descriptively by cross-tabulating the CPR self-assessment item against each objectively scored item and by counting respondents who selected the keyed answer on all four items; this count was used only to describe agreement with self-assessment, and these comparisons were not tested.

All inferential analyses were exploratory and hypothesis-generating. Associations between sociodemographic characteristics and receipt of CPR training, and between training status and each knowledge or practice item, were examined using Fisher’s exact test, the Fisher–Freeman–Halton exact test, and the independent-samples Mann–Whitney U test, as appropriate; *p*-values were not adjusted for multiple comparisons and are interpreted as descriptive indicators rather than confirmatory tests. For binary comparisons, unadjusted odds ratios (ORs) with 95% confidence intervals (CIs) are reported to convey the size of differences between self-selected groups, not training effects. To examine whether the sociodemographic associations with training were independent of one another, all sociodemographic variables were entered simultaneously into a multivariable binary logistic regression model with receipt of CPR training as the outcome; adjusted ORs with 95% CIs are reported in Table S2. Sparse categories were combined before modeling (marital status: single or ever married [married, separated, or widowed]; educational level: secondary or below, diploma, bachelor’s, postgraduate; occupation: student, government employee, private sector, homemaker, retired), and age was modeled per 10-year increment. Participant status (citizen vs. resident) was not analyzed because only one respondent was a resident. The model included 172 events for 11 estimated coefficients (approximately 16 events per coefficient); overall fit is summarized with the likelihood-ratio test and McFadden’s pseudo-R^2^. Two sensitivity analyses were performed: Firth penalized-likelihood logistic regression with profile-likelihood CIs [19], which reduces small-sample bias arising from sparse categories, and a model with educational level further collapsed into two categories (diploma or below vs. bachelor’s or above). Missing data were not imputed. All sociodemographic and outcome variables were complete for all 364 respondents. Item-level non-response was limited to the following: three respondents typed a free-text comment (e.g., “I don’t know”) instead of selecting an option in the multiple-response warning-sign item (analyzed *n* = 361); one of the 219 respondents reporting BLS training did not report the number of sessions (analyzed *n* = 218); and one of the 83 respondents who reported witnessing sudden death did not answer the follow-up item on the action taken (analyzed *n* = 82). All remaining blank fields reflect questionnaire skip logic rather than non-response. A two-sided *p*-value < 0.05 was considered statistically significant. Analyses were performed using IBM SPSS Statistics, Version 29.0 (IBM Corp., Armonk, NY, USA), and Python 3.11 (Python Software Foundation, Wilmington, DE, USA) with SciPy 1.17.

### 2.2. Ethical Considerations

The study was approved by the Research Ethics Committee of King Khalid University (approval no. ECM#2025-302; 16 February 2025). Participation was voluntary and anonymous. The informed consent form was presented to every participant as the first section of the electronic questionnaire, and only those who read it and recorded their agreement to take part were able to proceed to the study questions. The approval covered the complete questionnaire, including the exact wording of all items. No personal identifiers were collected. All procedures were conducted in accordance with the Declaration of Helsinki.

## 3. Results

A total of 364 respondents were analyzed after removal of two duplicate submissions (Section 2), exceeding the recruitment target of 315. The mean age was 30.41 ± 12.74 years (median 24.00 years, IQR 20.00–40.00). The sample was predominantly female (87.1%) and single (59.1%); most held a bachelor’s degree (67.9%), and 49.5% were students. All participants reported residence in the Aseer Region, and 74.7% lived in urban areas. This composition differs markedly from that of the regional adult population, so all percentages describe this self-selected sample only (Section 4.7). Sociodemographic details are presented in Table 2.

### 3.1. Awareness and Training Related to Basic Life Support

Most participants (90.9%) reported that they had heard of basic life support. Overall, 60.2% of the sample had received formal BLS training, and 47.3% had undergone CPR training. Of those who had received BLS training (N = 219), 54.8% received both theoretical and practical training, 38.8% theoretical training only, and 6.4% practical training only. The most common source of BLS information was healthcare workers (48.6%), followed by media and social media platforms (33.5%). Full details are presented in Table 3.

Responses to the combined warning-sign item, which are reported descriptively only (Section 2), and to the related assessment items are shown in Table 4. Among the 361 respondents who selected at least one option, the most frequently selected responses were cessation of blood circulation (60.1%), chest pain (58.2%), and loss of consciousness (52.4%). Assessment of unconsciousness was most commonly linked to “no response when called” (65.9%) and “no response when touched” (51.9%). Cessation of breathing was primarily identified by “no chest movement” (68.1%) and “no breathing sound” (47.0%). The most frequently selected sign of circulatory cessation was inability to feel a carotid pulse (59.6%).

### 3.2. Attitudes and Practices Toward Sudden Death and CPR

Most respondents (78.3%) selected “I haven’t seen that case.” Because this was a multiple-response item, four of them also selected at least one witnessed category; in total, 83 respondents (22.8%) selected at least one witnessed category, and 82 of these answered the follow-up item on the action taken. Among these 82 respondents, the most common response was calling an ambulance (42.7%), followed by asking others to call for help (37.8%). The most frequent interpretation of the questionnaire term “heart massage” (i.e., chest compressions) was “applying strong pressure to the chest at specific intervals” (42.0%), while 29.1% reported no knowledge of the term.

Reported willingness to perform CPR was highest for a family member (87.1%) and lower for a friend (62.4%), a neighbor (53.6%), a young person at the gym (46.2%), and a stranger at a shopping center (44.8%). The two items worded with stigmatizing labels are shown in Table 5 for transparency only (Section 2). When asked about a family member or friend collapsing, 55.8% selected calling an ambulance first and 34.9% selected beginning CPR; for a stranger, 70.6% selected calling an ambulance and 23.6% selected performing CPR. Details are in Table 5.

### 3.3. CPR Training, Self-Assessed and Objectively Scored Knowledge, and Concerns

Within this sample, 47.3% reported CPR training and 45.1% reported knowing how to perform CPR. Responses to the general BLS-training item and the CPR-training item were discordant for 85 respondents (23.4%): 66 reported BLS but not CPR training, 43 of whom described their BLS training as theoretical only, and 19 reported CPR but not BLS training. The most common training locations were universities (23.3%) and schools (22.7%). On the objectively scored items, 33.5% selected the 30:2 compression-to-ventilation ratio, 57.1% the middle of the chest as the compression site, 27.5% a rate of at least 100 compressions/min, and 45.3% a compression depth of 5–6 cm. Familiarity with the AED was reported by 53.8%.

Self-assessed and objectively scored knowledge diverged (descriptive comparison, not tested). Among the 164 respondents who reported knowing how to perform CPR, 51.8% selected the 30:2 ratio, 68.9% the middle of the chest, 40.9% a rate of at least 100/min, and 57.3% a depth of 5–6 cm; 34 (20.7%) selected the keyed answer on all four items, and 18 (11.0%) on none. Among the 200 respondents who reported not knowing how to perform CPR, 3 (1.5%) selected all four keyed answers. Of the 172 respondents reporting CPR training, 129 (75.0%) reported knowing how to perform CPR, compared with 35 of the 192 (18.2%) not reporting training.

The primary concern preventing CPR performance for friends/relatives was fear of making a mistake (58.2%), and for strangers the same concern dominated (53.3%). Additional concerns for strangers included fear of legal punishment (8.0%) and exposure to infectious disease (3.8%). Full data are presented in Table 6.

### 3.4. Association Between Sociodemographic Characteristics and Receipt of CPR Training

In bivariate analysis, receipt of CPR training was associated with age (*p* < 0.001), marital status (*p* < 0.001), and occupation (*p* < 0.001), but not with gender (*p* = 0.640), educational level (*p* = 0.167), or place of residence (*p* = 0.147). Trained participants were younger (mean 27.05 ± 10.66 years) than untrained participants (33.43 ± 13.68 years); single individuals reported higher training rates (58.1%) than married individuals (31.4%); and training was most common among private sector employees (62.5%) and students (60.0%) and least common among homemakers (14.5%) and retired individuals (15.8%). Participant status was not tested because only one respondent was a resident. These results are shown in Table 7.

In an exploratory multivariable model including all measured sociodemographic variables (Table S2), the lower odds of reported CPR training among homemakers than among students persisted (adjusted OR 0.17, 95% CI 0.06–0.47), whereas the crude associations with age and marital status did not; the homemaker estimate was similar in both sensitivity analyses. The model explained little of the variation in training (McFadden pseudo-R^2^ = 0.11), so it does not identify the determinants of training (Section 4.5).

### 3.5. Association Between Reported CPR Training and Knowledge and Practice Items

**Figure 1:**
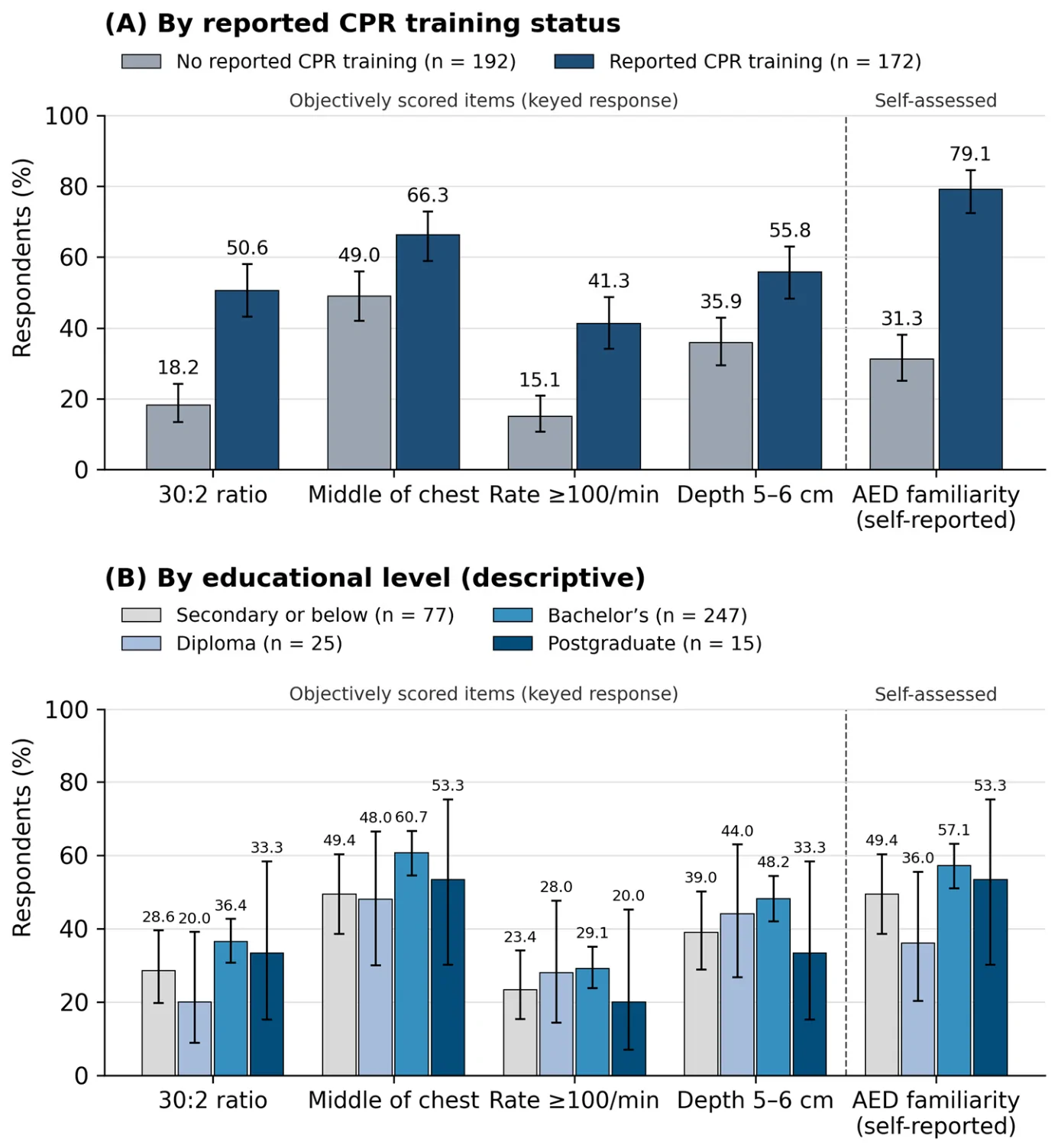
Item-level proportions for the four objectively scored CPR knowledge items and the self-reported AED familiarity item (**A**) by reported CPR training status and (**B**) by educational level. For the objectively scored items, the keyed responses were the 30:2 compression-to-ventilation ratio, the middle of the chest as the compression site, a rate of at least 100 compressions/min, and a depth of 5–6 cm; for the AED item, the proportion reporting familiarity with the automated external defibrillator (AED) is shown. Error bars show Wilson 95% confidence intervals. Panel A corresponds to Table 8 (all items, *p* < 0.001). Panel B is descriptive and was not formally tested; the diploma (*n* = 25) and postgraduate (*n* = 15) estimates are imprecise.

Respondents reporting CPR training selected the keyed answer more often than those not reporting training on each of the four objectively scored items (Table 8 and Figure 1A; percentages within each training group; unadjusted ORs): the 30:2 ratio (50.6% vs. 18.2%; OR 4.59, 95% CI 2.86–7.37), the middle of the chest (66.3% vs. 49.0%; OR 2.05, 95% CI 1.34–3.13), a rate of at least 100/min (41.3% vs. 15.1%; OR 3.95, 95% CI 2.40–6.50), and a depth of 5–6 cm (55.8% vs. 35.9%; OR 2.25, 95% CI 1.48–3.43). The differences were therefore largest for the ratio and rate. For the rate item, part of the difference reflected the “I don’t know” option, selected by 59.9% of respondents not reporting training and by 21.5% of those reporting training; among respondents who chose a numerical option, 52.6% (71/135) of those reporting training and 37.7% (29/77) of those not reporting training selected at least 100/min. Self-reported AED familiarity was also more common among respondents reporting training (79.1% vs. 31.3%; OR 8.31, 95% CI 5.16–13.40). By educational level, the proportions were numerically highest among respondents with a bachelor’s degree for all five items, but the differences were modest, and the diploma (*n* = 25) and postgraduate (*n* = 15) groups were small; these proportions are shown descriptively and were not formally tested (Figure 1B).

These comparisons are between self-selected groups (Section 4.2). Complete results are presented in Table 8.

## 4. Discussion

In this self-selected online sample, the constructs examined were only loosely aligned: almost all respondents had heard of BLS, about half reported CPR training or considered themselves able to perform CPR, and only one in five of the latter selected the keyed answer on all four objectively scored items. Reported training was associated with more keyed answers, as is well established, but the size of the difference varied across items. Beyond confirming this association, the study contributes a within-sample separation of awareness, self-assessed knowledge, and objectively scored knowledge; item-level evidence on which technical parameters were least often answered correctly, including by trained respondents, and on how response options may have shaped these estimates; and an openly shared dataset from a region without a previous dedicated survey. All interpretations apply to respondents only, within the limitations set out in Section 4.7.

### 4.1. Awareness, Self-Assessed Knowledge, and Objectively Scored Knowledge

Having heard of BLS indicates exposure to the term, not the ability to act; it was nearly universal among both students and nonstudents (91.7% and 90.2%), probably because all respondents shared characteristics associated with participation in online, health-related surveys, yet fewer than half reported CPR training. Self-assessment added a further layer. Three-quarters of respondents reporting training considered themselves able to perform CPR, but among all who did so, about half selected the 30:2 ratio, fewer than half selected the rate option, and 20.7% selected the keyed answer on all four items. Self-assessed ability may reflect confidence or general familiarity rather than recall of specific parameters. Similarly, the AED item recorded familiarity with the device, so the large difference between training groups on this item (Table 8) shows that trained respondents more often recognized the device, not that they knew how to use it. Prior Saudi surveys have reported limited practical knowledge despite high awareness [11,14]; the present data show this gap within the same respondents and at the item level, which suggests that awareness and self-assessment are weak proxies for knowledge.

### 4.2. Reported Training and Item-Level Knowledge

Training itself was not a uniform construct: the general BLS training and CPR training items were answered discordantly by 23.4% of respondents, and about half of those whose BLS training had been theoretical only did not report CPR training. Because course content, practical components, certification, and recency were not recorded, reported training is best regarded as a heterogeneous self-reported exposure rather than a defined intervention.

The association with reported training was modest for the compression site and depth, which respondents without training often answered correctly (49.0% and 35.9%), and larger for the ratio and rate, which few of them selected (18.2% and 15.1 %). The ratio and rate are numerical conventions that are hard to infer without instruction, yet they were also the parameters most often missed by trained respondents: 49.4% did not select 30:2, and 58.7% did not select the rate option. The most frequently selected ratio overall was 15:2 (37.4%, including 31.4% of trained respondents); because 15:2 preceded the current adult recommendation of 30:2 [20], its selection may reflect outdated information rather than an absence of information. Knowledge decay after training [21], differences in course content or recency, and item construction (Section 4.3) may all contribute. These parameter-specific gaps, rather than the overall contrast between trained and untrained respondents, are the more informative targets for training evaluations.

The comparisons also partly reflected willingness to commit to an answer. For the rate item, most respondents not reporting training selected “I don’t know”, and among respondents who chose a numerical option the difference between groups was smaller (52.6% vs. 37.7%). Differences on knowledge items may therefore combine differences in knowledge with differences in confidence or response style.

Most importantly, these are comparisons between self-selected groups: training was self-reported rather than assigned, and the item-level ORs are unadjusted. People who seek training, or whose studies or work require it, may differ from others in health literacy, health-science education or healthcare exposure, interest in emergency care, and previous emergency experiences, each of which could independently increase the likelihood of answering correctly. Reported training was concentrated among students and private-sector employees and was uncommon among homemakers and retired respondents (Table 7), whereas field of study, healthcare employment, and the circumstances in which training was obtained were not recorded. The associations may therefore reflect the characteristics of people who choose, or are required, to undergo training rather than effects of training itself, and they do not show that respondents could perform CPR; estimating training effects requires longitudinal or experimental designs with observed performance.

### 4.3. Measurement Considerations and Lessons for Instrument Development

Because the items were not validated, the observed differences may partly reflect item wording and response options rather than knowledge alone. The rate options (“at least 50”, “at least 100”, and “at least 150” compressions/min) did not include the recommended 100–120/min [16], so the keyed option was only the closest available choice. The depth options paired distances with descriptions of force, labeling the keyed 5–6 cm option “moderate pressure” and the 6–10 cm option “strong pressure”; the latter was selected by 15.7% of trained respondents, perhaps because they had been taught to push hard. The site options did not distinguish the center of the chest from the lower half of the sternum, where the hands are recommended to be placed [16]; the ratio item did not specify an adult or single-rescuer context; and the AED item recorded familiarity rather than tested knowledge. Other items had similar problems: the collapse scenarios required a choice between calling an ambulance and starting compressions, whereas bystanders are expected to call for help and start compressions without delay [16]; the warning-sign item combined myocardial infarction and cardiac arrest features, although these emergencies call for different bystander responses; and the two stigmatizing willingness items changed social proximity, hygiene cues, perceived infection risk, and moral judgment simultaneously, so their responses may reflect reactions to the wording rather than willingness to help.

These problems share a common origin. Expert review and a small pilot addressed relevance, comprehensibility, and completion time, but no cognitive interviewing examined how respondents interpreted items and response options, and no community stakeholders reviewed the wording. The stigmatizing items show this most clearly: adapted from prior studies (Section 2) and approved as part of the complete questionnaire, they were not identified as inappropriate by either expert review or pilot testing, and the authors regard their inclusion as a shortcoming of the instrument-development process, not merely of wording. Because several items were adapted from earlier regional surveys [5,6,11], similar problems may affect other instruments, and response options should be examined before correct-response percentages or composite scores are compared across studies. Future instruments should be developed with cognitive interviewing; involvement of community stakeholders, including members of the groups described in scenario items; respectful, person-first wording; scenarios that separate social proximity from perceived infection risk, with item order randomized or balanced where feasible; keyed options aligned with current guidelines; separate measures of myocardial infarction and cardiac arrest recognition; and formal psychometric evaluation before deployment.

### 4.4. Willingness and Concerns

Among the neutrally worded categories, reported willingness to perform CPR declined with social distance, from 87.1% for a family member to 44.8% for a stranger at a shopping center, consistent with previous reports of lower willingness toward socially distant recipients [6,12,17,18]. Fear of making a mistake was the most common concern for both relatives and strangers, as in several previous surveys [12,14,22], whereas concern about legal consequences was selected more often for strangers (8.0%) than for relatives (1.9%). These are hypothetical intentions and concerns in this sample rather than observed bystander behavior; they suggest that training content addressing fear of error and information on the legal position of lay rescuers could be evaluated in future programs.

### 4.5. Sociodemographic Correlates of Reported CPR Training

Reported training was associated with age, marital status, and occupation in bivariate analysis, but after mutual adjustment only the lower odds among homemakers persisted, and the model explained little of the variation in training (McFadden pseudo-R^2^ = 0.11; Table S2). Its main message is therefore that the measured sociodemographic characteristics, including educational level, which was not independently associated with training, account for little of who reported training; determinants of training have not been identified. Unmeasured factors are likely to matter more and could confound the occupational estimate; they include income, which was associated with CPR training in a national survey in the United States [10] and with BLS knowledge scores in a recent Saudi survey from the Jazan Region [23], as well as healthcare affiliation, employer requirements, course availability and cost, caregiving responsibilities, and motivation. One hypothesis worth testing is that schools, universities, and workplaces provide routes to training that are less available to other adults; outreach through primary healthcare centers, women’s organizations, and retiree associations could be evaluated as a way to reach them.

### 4.6. Comparison with Previous Studies

Table 9 summarizes selected regional and international studies as descriptive context. Because the studies differ in populations, sampling, questionnaire wording, definitions of training, and knowledge scoring, their figures are not directly comparable with those of the present study; in particular, the 90.9% awareness figure reflects a self-selected online sample (Section 4.7) rather than an Aseer advantage. Qualitatively, other studies also reported better knowledge among trained respondents [2,22] and fear of making a mistake as a common barrier [12,14,22].

### 4.7. Strengths and Limitations

The study has three strengths. It provides a de-identified participant-level dataset with a variable dictionary (Table S1), from which every reported frequency, odds ratio, and model estimate can be reproduced; it separates self-reported, self-assessed, and objectively scored constructs within the same respondents and analyzes knowledge at the item level; and it reports the weaknesses of its instrument in enough detail to inform the design of future surveys in an under-studied region.

The limitations apply to every result reported above. First, convenience recruitment through an open online link produced a self-selected sample that was 87.1% female, 49.5% student, and 67.9% bachelor’s educated; people with internet access, health interest, university connections, or links to the distributing networks were more likely to participate. The findings describe respondents only and should not be generalized to the Aseer adult population; awareness and training estimates may be inflated, as suggested by the higher proportions reporting CPR training among students than among nonstudents (60.0% vs. 34.8%) and among respondents with a bachelor’s or postgraduate degree than among those with less education (50.8% vs. 38.2%). The recruitment target was derived from a formula that assumes probability sampling, so no population margin of error applies, and small numbers in several male, older, occupational, and educational subgroups reduce precision. Second, the questionnaire was expert reviewed and pilot-tested for comprehensibility but was not psychometrically validated and did not undergo cognitive interviewing or stakeholder review; the technical items had poor internal consistency, possibly reflecting their brevity and heterogeneous content, the validity of individual items has not been established, and several items had the construction problems described in Section 4.3. Third, all training, knowledge, and behavioral responses were self-reported; no certification records or observed CPR skills were assessed, course content and recency were not recorded, and recall and social-desirability bias are possible. Fourth, cross-sectional comparisons between self-selected training groups cannot distinguish effects of training from characteristics of the people who seek it. Fifth, the exploratory regression model explained little of the variation in training, and important potential determinants were not measured. Finally, online-survey controls were limited: view, participation, and completion rates could not be determined, no technical safeguard against repeat participation was used, and two duplicate submissions were identified only through post hoc screening and were removed.

### 4.8. Implications for Future Research and Practice

These findings generate priorities for evaluation rather than evidence that any intervention will be effective. Representative studies should use probability-based or stratified sampling and an instrument developed as described in Section 4.3, and should measure awareness, self-assessed knowledge, objectively scored knowledge, and observed skills as separate outcomes. Training evaluations should document course content, practical components, certification, instructor qualifications, recency, and refresher exposure, and should assess retention of specific parameters such as the compression-to-ventilation ratio and compression rate, which were the least often answered correctly by trained respondents in this sample. Community programs that aim to reach adults outside school, university, and workplace pathways should be evaluated with equitable reach and objective outcomes.

## 5. Conclusions

This exploratory study of a self-selected online sample of Aseer residents found that self-reported awareness of BLS and self-assessed ability to perform CPR were often not matched by objectively scored knowledge: only one in five respondents who reported knowing how to perform CPR selected the keyed answer on all four objectively scored items. Respondents reporting CPR training selected keyed answers more often, most markedly for the compression-to-ventilation ratio and compression rate, yet these were also the parameters most often missed by trained respondents. Measured sociodemographic characteristics explained little of the variation in reported training.

Because the sample was self-selected, the questionnaire was not validated, and training was self-reported rather than assigned, these results do not estimate regional prevalence, show that training caused better knowledge, or measure CPR competence (Section 4.7). Their value lies in distinguishing constructs that are often conflated, identifying specific knowledge gaps and instrument weaknesses, and providing an openly shared dataset to guide representative studies with validated, respectfully worded instruments and objective assessment of CPR skills.

## Figures and Tables

**Table 1:** Constructs Examined, Their Operational Definitions, and the Corresponding Questionnaire Items.

Construct	Operational Definition	Questionnaire Item(s) and Response Format	Use in Analysis
**Awareness**	Self-reported familiarity with BLS	Having heard of BLS (yes/no); main source of information (single choice)	Descriptive (Section 3.1)
**Training**	Self-reported receipt of instruction	BLS training (yes/no), with its type and number of sessions; CPR training: training on “this topic (life-saving basics)”, asked directly after the CPR self-assessment item (yes/no), with training location	Descriptive (Section 3.1 and Section 3.3); CPR training is the grouping variable in Section 3.4 and Section 3.5
**Self-assessed knowledge**	Respondents’ own judgment of their knowledge	Knowing how to perform CPR (yes/no); AED item: what respondents know about the “electric shock” device (Yes; No, I don’t know)	Descriptive and compared with objectively scored items (Section 3.3); AED item by training (Section 3.5)
**Objectively scored knowledge**	Selection of the keyed, guideline-concordant option	Single-best-answer items: compression-to-ventilation ratio (keyed 30:2), compression site (middle of the chest), rate (at least 100/min), and depth (5–6 cm)	Each item analyzed individually (Section 3.3 and Section 3.5)
**Recognition-related responses**	Signs selected as indicating an emergency or loss of consciousness, breathing, or circulation	Combined heart attack/cardiac arrest warning-sign item and three assessment items (multiple responses)	Descriptive only (Section 3.1)
**Attitudes**	Reported willingness to perform CPR and concerns about doing so	Willingness across recipient categories (multiple responses); concerns for relatives and for strangers (single choice)	Descriptive only (Section 3.2 and Section 3.3); two stigmatizing willingness items not interpreted
**Reported and intended practice**	Actions taken or intended in an emergency	Action taken when sudden death was witnessed; first action in two hypothetical collapse scenarios; preferred life-saving method; meaning of “heart massage”	Descriptive (Section 3.2 and Section 3.3); life-saving method by training (Section 3.5)

BLS = basic life support; CPR = cardiopulmonary resuscitation; AED = automated external defibrillator. Item wording is summarized from the variable dictionary (Table S1). Keyed options were those closest to current adult recommendations; no rate option matched the recommended 100–120 compressions/min.

**Table 2:** Sociodemographic Characteristics of the Study Participants (N = 364).

		N	%
**Age (years)**	Mean ± SD	30.41 ± 12.74	
	Median (IQR)	24.00 (20.00–40.00)	
**Gender**	Female	317	87.1%
	Male	47	12.9%
**Marital Status**	Married	140	38.5%
	Separated	5	1.4%
	Single	215	59.1%
	Widowed	4	1.1%
**Educational Level**	Postgraduate studies	15	4.1%
	Bachelor’s	247	67.9%
	Diploma	25	6.9%
	Secondary	70	19.2%
	Primary	5	1.4%
	Preparatory	2	0.5%
**Occupation**	Government job	79	21.7%
	Homemaker	62	17.0%
	Private sector	24	6.6%
	Retired	19	5.2%
	Student	180	49.5%
**Residential Area**	Aseer Region	364	100.0%
**Place of Residence**	City	272	74.7%
	Village	92	25.3%
**Participant Status**	Citizen	363	99.7%
	Resident	1	0.3%

**Table 3:** BLS Awareness, Training, and Sources of Information.

		N	%
**Heard of basic life support**	No	33	9.1%
	Yes	331	90.9%
**Received BLS training**	No	145	39.8%
	Yes	219	60.2%
**Type of BLS training (N = 219)**	Both (theoretical and practical)	120	54.8%
	Theoretical	85	38.8%
	Practical	14	6.4%
**Number of training sessions (N = 218)**	Once	128	58.7%
	Twice	55	25.2%
	Three times or more	35	16.1%
**Source of information**	Healthcare workers	177	48.6%
	Media outlets/social media platforms	122	33.5%
	Friends and relatives	33	9.1%
	Books and reading materials	32	8.8%

**Table 4:** Participants’ Responses to the Combined Heart Attack/Cardiac Arrest Warning-Sign Item and Related Assessment Items (N = 364 unless stated). Multiple responses were allowed. The warning-sign item combined myocardial infarction symptoms with cardiac arrest features, does not measure cardiac arrest recognition, and is reported descriptively only; its percentages use *n* = 361, because three respondents typed a free-text comment (e.g., “I don’t know”) instead of selecting an option.

		N	%
**Signs of heart attack/cardiac arrest (*n* = 361)**	Cessation of blood circulation	217	60.1%
	Chest pain	210	58.2%
	Loss of consciousness	189	52.4%
	Cessation of breathing	143	39.6%
	Immobility	128	35.5%
	Cyanosis	122	33.8%
	Weakness	99	27.4%
	Nausea	89	24.7%
	Respiratory arrest	51	14.1%
**Assessment of consciousness**	No response when called	240	65.9%
	No response when touched	189	51.9%
	It doesn’t move at all	135	37.1%
	I don’t know	55	15.1%
**Identification of breathing cessation**	No chest movement	248	68.1%
	No breathing sound	171	47.0%
	No air from mouth	151	41.5%
	No vapor on mirror	78	21.4%
	I don’t know	42	11.5%
**Determination of cessation of blood circulation**	Inability to feel a carotid pulse	217	59.6%
	Signs of poor circulation	213	58.5%
	Inability to feel a pulse in the wrist veins	174	47.8%

**Table 5:** Attitudes and Practices Toward Sudden Death and CPR. Multiple responses were allowed for the witnessed sudden death, action taken, and willingness items. Percentages use N = 364 except for the action taken (*n* = 82). The last two willingness items used stigmatizing questionnaire wording that the authors do not endorse; they are reported for transparency only and are not interpreted as estimates of willingness (Section 2).

		N	%
**Witnessed sudden death**	I haven’t seen that case	285	78.3%
	A member of my family	48	13.2%
	Friend or acquaintance	28	7.7%
	Stranger	19	5.2%
**Action taken during sudden death (N = 82)**	Called an Ambulance	35	42.7%
	Asked others to call for help	31	37.8%
	CPR	25	30.5%
	Mouth-to-mouth resuscitation	25	30.5%
	Called for help by phone/mobile	13	15.9%
	Watched the situation and left	13	15.9%
**Meaning of “heart massage”**	Applying strong pressure to the chest at specific intervals	153	42.0%
	Apply pressure to the heart immediately after exposing the chest area	81	22.3%
	Massaging the heart at specific intervals	73	20.1%
	Massaging the heart immediately after exposing the chest area	45	12.4%
	I have no idea what it means	106	29.1%
**Willingness to give CPR to**	A family member	317	87.1%
	A friend	227	62.4%
	A neighbor	195	53.6%
	The young man/woman at the gym	168	46.2%
	Any stranger at the supermarket or shopping center	163	44.8%
	A person described in the questionnaire as “dirty and unclean” at a bus stop	136	37.4%
	A person described in the questionnaire as using or being dependent on drugs, hashish, or prescription medications	107	29.4%
**Response to family/friend collapse**	Call an ambulance (997)	203	55.8%
	I begin by performing a heart massage	127	34.9%
	I contact people I know or call for help	28	7.7%
	I just observe and then leave.	6	1.6%
**Response to stranger collapse**	Call an ambulance (997)	257	70.6%
	I begin with a heart massage.	86	23.6%
	I contact people I know or call to ask for help	14	3.8%
	I just observe, then I leave.	7	1.9%

**Table 6:** CPR Training, Knowledge, and Concerns. N = 364 unless stated. Knowing how to perform CPR and AED familiarity are self-assessed; the ratio, site, rate, and depth items are objectively scored.

		N	%
**Concerns preventing CPR (friends/relatives)**	Making a mistake	212	58.2%
	Causing harm to body organs	40	11.0%
	Causing a bone fracture	35	9.6%
	Cardiac arrest	13	3.6%
	Punishment due to prevailing legislation/laws	7	1.9%
	Exposure to an infectious disease	4	1.1%
	Contamination from blood and vomit	1	0.3%
	Other	52	14.3%
**Concerns preventing CPR (strangers)**	Making a mistake	194	53.3%
	Causing harm to body organs	33	9.1%
	Punishment due to prevailing legislation/laws	29	8.0%
	Causing a bone fracture	23	6.3%
	Cardiac arrest	18	4.9%
	Exposure to an infectious disease	14	3.8%
	Contamination from blood and vomit	10	2.7%
	Other	43	11.8%
Knows how to perform CPR (self-assessed)	No	200	54.9%
	Yes	164	45.1%
**Received CPR training**	No	192	52.7%
	Yes	172	47.3%
**Training location (N = 172)**	At the university	40	23.3%
	At school	39	22.7%
	Trainer from the Ministry of Health	36	20.9%
	CPR training institutions	29	16.9%
	Media, television, internet	12	7.0%
	Training course from the municipality	4	2.3%
	Driving schools	3	1.7%
	In the workplace	2	1.2%
	In military service	2	1.2%
	At the sports club	1	0.6%
	Other	4	2.3%
**Life-saving method used**	I perform oral resuscitation and CPR together.	114	31.3%
	Massage the heart	86	23.6%
	Opening the airway	47	12.9%
	I perform oral resuscitation (mouth-to-mouth).	26	7.1%
	Breathing control	4	1.1%
	I don’t know	87	23.9%
**Correct compression–ventilation ratio**	5:1	106	29.1%
	15:2	136	37.4%
	30:2	122	33.5%
**Compression site**	Middle chest	208	57.1%
	Upper chest	73	20.1%
	Lower chest	51	14.0%
	Other	32	8.8%
**Compression rate**	I don’t know	152	41.8%
	At least 100 times/minute	100	27.5%
	At least 50 times/minute	74	20.3%
	At least 150 times/minute	38	10.4%
**Compression depth**	1–2 cm	87	23.9%
	5–6 cm	165	45.3%
	6–10 cm	43	11.8%
	Maximum force	15	4.1%
	Other	54	14.8%
Familiarity with the automated external defibrillator (AED; self-assessed)	Yes	196	53.8%
	No, I don’t know.	168	46.2%

**Table 7:** Association Between Sociodemographic Characteristics and Receipt of CPR Training. Percentages are calculated within each sociodemographic category (row percentages).

		Received CPR Training				
		No (N = 192)		Yes (N = 172)		*p* Value
		N	Row %	N	Row %	
**Age (years)**	Mean ± SD	33.43 ± 13.68		27.05 ± 10.66		<0.001 *
	Median (IQR)	30.50 (20.00–45.00)		23.00 (20.00–28.00)		
**Gender**	Female	169	53.3%	148	46.7%	0.640
	Male	23	48.9%	24	51.1%	
**Marital status**	Married	96	68.6%	44	31.4%	<0.001 *
	Separated	2	40.0%	3	60.0%	
	Single	90	41.9%	125	58.1%	
	Widowed	4	100.0%	0	0.0%	
**Educational level**	Bachelor’s	123	49.8%	124	50.2%	0.167
	Diploma	17	68.0%	8	32.0%	
	Postgraduate studies	6	40.0%	9	60.0%	
	Preparatory	2	100.0%	0	0.0%	
	Primary	2	40.0%	3	60.0%	
	Secondary	42	60.0%	28	40.0%	
**Occupation**	Government job	42	53.2%	37	46.8%	<0.001 *
	Homemaker	53	85.5%	9	14.5%	
	Private sector	9	37.5%	15	62.5%	
	Retired	16	84.2%	3	15.8%	
	Student	72	40.0%	108	60.0%	
**Place of residence**	City	137	50.4%	135	49.6%	0.147
	Village	55	59.8%	37	40.2%	

Fisher’s Exact test; Fisher–Freeman–Halton Exact test; Independent-Samples Mann–Whitney U test. * *p* < 0.05, significant. Participant status (citizen, *n* = 363; resident, *n* = 1) was not tested because the resident category contained a single respondent.

**Table 8:** Association Between Reported CPR Training and Knowledge and Practice Items. Percentages are calculated within each training group (column percentages). The AED item records self-reported familiarity; the ratio, site, rate, and depth items are objectively scored.

		Received CPR Training				
		No (N = 192)		Yes (N = 172)		*p* Value
		N	% of Group	N	% of Group	
**Life-saving method used**	I perform oral resuscitation and CPR together.	35	18.2%	79	45.9%	<0.001 *
	I don’t know	77	40.1%	10	5.8%	
	Massage the heart	44	22.9%	42	24.4%	
	Opening the airway	18	9.4%	29	16.9%	
	I perform oral resuscitation (mouth-to-mouth).	17	8.9%	9	5.2%	
	Breathing control	1	0.5%	3	1.7%	
**Correct compression–ventilation ratio**	5:1	75	39.1%	31	18.0%	<0.001 *
	15:2	82	42.7%	54	31.4%	
	30:2	35	18.2%	87	50.6%	
**Compression site**	Middle chest	94	49.0%	114	66.3%	<0.001 *
	Upper chest	48	25.0%	25	14.5%	
	Lower chest	22	11.5%	29	16.9%	
	Other	28	14.6%	4	2.3%	
**Compression rate**	I don’t know	115	59.9%	37	21.5%	<0.001 *
	At least 50 times/minute	34	17.7%	40	23.3%	
	At least 100 times/minute	29	15.1%	71	41.3%	
	At least 150 times/minute	14	7.3%	24	14.0%	
**Compression depth**	1–2 cm	56	29.2%	31	18.0%	<0.001 *
	5–6 cm	69	35.9%	96	55.8%	
	6–10 cm	16	8.3%	27	15.7%	
	Maximum force	8	4.2%	7	4.1%	
	Other	43	22.4%	11	6.4%	
AED familiarity (self-assessed)	Yes	60	31.3%	136	79.1%	<0.001 *
	No, I don’t know.	132	68.8%	36	20.9%	

Fisher’s Exact test; Fisher–Freeman–Halton Exact test. * *p* < 0.05, significant.

**Table 9:** Comparison of the Current Study with Selected Regional and International BLS/CPR Studies.

Study (Year)	Country/Region	N	BLS Awareness	BLS/CPR Training	CPR Knowledge	Key Reported Finding
Current Study (2025)	Aseer online sample, Saudi Arabia	364	90.9%	BLS: 60.2%; CPR: 47.3%	45.1% self-assessed	Fear of mistakes top barrier (58.2%); reported training was associated with higher keyed-response proportions on all four objectively scored items and with more frequent self-reported AED familiarity (*p* < 0.001).
Alghamdi et al. (2024) [14]	5 Regions, Saudi Arabia (Nationwide)	992	Not reported separately	41.4%	Mean score 9.64/20 (48%); 47% poor knowledge	Younger, educated, and healthcare-affiliated participants had higher knowledge. Fear of error top barrier (63.1%).
Sayed et al. (2024) [12]	Jazan Region, Saudi Arabia	480	Not reported	36.0% CPR training	Education & student status = higher scores	Only 36% received CPR training. Fear of making a mistake was top barrier (70.63%). Willingness to perform CPR exceeded 90% for family members and was 65.0% for friends. Low CPR knowledge among non-medically trained residents.
Abbas et al. (2021) [11]	Jeddah, Saudi Arabia	740	Not reported	Not reported	Mean 5.3/15 (inadequate)	Predominant age group 18–39 years. Inadequate overall knowledge despite moderate sample literacy.
Almutairi et al. (2023) [7]	Al-Majma’ah, Saudi Arabia	671	Moderate understanding of cardiac arrest & AED concepts	Not reported	Gaps in correct initial steps	Age significantly associated with BLS knowledge (*p* = 0.014). Gender influenced attitudes. Confusion about correct first-response steps.
Alrasheedi et al. (2022) [2]	Al-Qassim Region, Saudi Arabia	414	Not reported	19.8% CPR training	25.5% adequate CPR knowledge	Participants had relatives with heart disease. CPR-trained participants had better knowledge (OR 1.82); lack of knowledge about available courses was the main reason for not training (60.5%).
Al-Riyami et al. (2020) [24]	Muscat, Oman	1080	Not reported	Not reported	52.9% willing to perform CPR on stranger	Younger age, educational status, and CPR course attendance were associated with willingness. Lack of knowledge (47%) and fear of harm (29%) were main barriers.
Jarrah et al. (2018) [22]	Jordan (Cross-sectional)	300	Not reported	Not reported	No composite knowledge score reported	Trained participants answered individual knowledge items correctly more often than untrained participants. Willingness highest for family members (88.3%) vs. friends (36.7%) and neighbors (30.0%). Fear of making a mistake was the primary barrier (61.7% family; 59.0% strangers). Only 29% had received CPR training.
Blewer et al. (2017) [10]	United States (National)	9022	Not reported	65% ever trained; 18% currently trained	Not reported	Older age, lower education, and lower income = reduced CPR training likelihood.
Yan et al. (2020) [18]	China (National)	99,186	Not reported	37.6% previous CPR training	21.5% familiar with AED	Overall, 73.4% were willing to attend CPR training; willingness was independently associated with female sex, higher education, and lower income.

Note: The studies differ in population, sampling method, instrument, and outcome definitions; values are shown for descriptive context only and are not directly comparable. N = sample size; BLS = basic life support; CPR = cardiopulmonary resuscitation; AED = automated external defibrillator.

## Data Availability

The de-identified participant-level dataset and variable dictionary supporting the findings of this study are provided in the Supplementary Materials (Table S1). Further information is available from the corresponding author on reasonable request.

## Supplementary Materials

The following supporting information can be downloaded at: https://www.mdpi.com/article/10.3390/healthcare14193311/s1: Table S1, De-identified participant-level dataset (N = 364) with the accompanying variable dictionary; Table S2, Exploratory multivariable logistic regression of sociodemographic characteristics and reported CPR training, with sensitivity analyses.
