# Supplementary material for: Basic Life Support Awareness, Knowledge, and Attitudes Among a Self-Selected Online Sample in the Aseer Region, Saudi Arabia: An Exploratory Cross-Sectional Survey
Source: Healthcare (Basel). 2026 Oct 4;14(19):3311. doi: 10.3390/healthcare14193311 (PMC13665039; doi:10.3390/healthcare14193311)
Supplement: Supplementary file 1 [file healthcare-14-03311-s001.zip › healthcare-4559289-supplementary.pdf]

Supplementary Materials

Manuscript healthcare-4559289: "Basic Life Support Awareness, Knowledge, and Attitudes among a Self-Selected Online Sample in the Aseer Region, Saudi Arabia: An Exploratory Cross-Sectional Survey". Table S1 (the de-identified participant-level dataset with its variable dictionary) is provided as a separate spreadsheet file.

Table S2. Exploratory Multivariable Logistic Regression of Sociodemographic Characteristics and Reported CPR Training (N = 364).

Variable	Category	Adjusted OR	95% CI	p-value
**Age**	per 10-year increase	0.82	0.55–1.23	0.343
**Gender**	Female	Reference		
	Male	1.18	0.58–2.37	0.652
**Marital status**	Single	Reference		
	Ever married (married/separated/widowed)	0.98	0.38–2.56	0.970
**Educational level**	Secondary or below	Reference		
	Diploma	0.89	0.29–2.75	0.845
	Bachelor's	1.19	0.65–2.18	0.570
	Postgraduate	2.40	0.67–8.54	0.177
**Occupation**	Student	Reference		
	Government employee	0.80	0.30–2.11	0.646
	Private sector	1.30	0.48–3.53	0.611
	Homemaker	0.17	0.06–0.47	0.001*
	Retired	0.23	0.04–1.36	0.106
**Place of residence**	City	Reference		
	Village	1.00	0.57–1.73	0.995

Adjusted odds ratios (ORs) with Wald 95% confidence intervals (CIs) from a single multivariable binary logistic regression model containing all listed variables simultaneously (N = 364; 172 respondents reporting CPR training; 11 estimated coefficients). Model likelihood-ratio $p < 0.001$; McFadden pseudo-R^2 = 0.11. Separated (n = 5) and widowed (n = 4) respondents were combined with married respondents because of sparse numbers. The model is exploratory and hypothesis-generating; it explained little of the variation in reported training and does not identify determinants of training. *$p < 0.05$, significant.

Sensitivity analyses: in Firth penalized-likelihood logistic regression (reference [23] of the main text), the OR was 0.18 (95% profile-likelihood CI 0.06–0.48) for homemakers and 0.27 (0.05–1.36) for retired respondents; with educational level collapsed into two categories (diploma or below vs. bachelor's or above), the adjusted OR for homemakers was 0.18 (95% CI 0.06–0.49).
